# Physical activity and diet modify the sex specific association between neurotrophin single nucleotide polymorphisms and insomnia in late‐life

**DOI:** 10.1002/alz.087747

**Published:** 2025-01-09

**Authors:** Hector L. Gonzalez, Sarah Schwartz, Gail B. Rattinger, Yin Liu, Mikaela Drewel, Maddy Mills, Mona Buhusi, JoAnn T. Tschanz

**Affiliations:** ^1^ Utah State University, Logan, UT USA; ^2^ Binghamton University, Binghamton, NY USA

## Abstract

**Background:**

Sleep disturbance is common in older adults at prevalence rates ranging between 30 ‐ 50% in the United States. Insomnia in late life is influenced by a wide array of genetic and lifestyle factors, some of which exhibit sex‐dependent effects. This study examined whether selected single nucleotide polymorphisms (SNPs) related to the neurotrophin, brain‐derived neurotrophic factor (BDNF), or its receptors and lifestyle factors, and their interactions were associated with the increased risk for sleep disturbance in older males and females.

**Method:**

The Cache County Study on Memory and Aging, a longitudinal, population study, enrolled 4,057 cognitively healthy participants [56.7% female; mean (SD) age = 74.78 (6.75) years]. Sleep disturbance (39% endorsed) was determined by self‐report of insomnia. Adherence to the Mediterranean diet and physical activity (sedentary, light, moderate and vigorous) were based on mail‐in questionnaires. The following SNPs were examined as predictors of sleep disturbance in separate logistic regression models for males and females: rs6265 (BDNF Val66Met), rs56164415 (BDNF C270T), rs2289656 (NTRK2 receptor TrkB), and rs2072446 (NGFR p75NTR). Covariates included age, body mass index, education, APOE ε4 status, and depression.

**Result:**

SNPs for BDNF or its receptors were not associated with sleep disturbance. Males experienced an 18% reduced odds of sleep disturbance with increasing levels of physical activity (OR (95%CI) = 0.83 [0.71; 0.97]; *p* = .023), whereas greater adherence to the Mediterranean diet was associated with an 8% higher odds of reporting sleep disturbance (OR [95%CI] = 1.08 [1.00; 1.18]; *p* = .047). Among females a trend for an interaction (*p* = .088) between Val66Met and physical activity indicated those with the minor allele who engaged in sedentary‐to‐light physical activity exhibited a 45% higher risk of sleep disturbance compared to those with the minor allele and moderate‐to‐vigorous physical activity (*p* = .026).

**Conclusion:**

Lifestyle factors of physical activities and diet affect the BDNF gene‐insomnia association differently for older males and females. Future studies may further explore interventions affecting BDNF levels (e.g., physical activity) as one potential way to treat insomnia in older adults.

